# Prognostic Significance of HMGA1 in Hepatocellular Carcinoma: Implications for Tumor Progression and Targeted Therapy

**DOI:** 10.34172/aim.34456

**Published:** 2025-10-01

**Authors:** Tianhao Tong, Bin Cheng, Wenhui Gao, Renyi Yang, Huiying Jian, Jingting Zhang, Zhuo Liu, Puhua Zeng

**Affiliations:** ^1^Cancer Research Institute of Hunan Academy of Traditional Chinese Medicine, Hunan Academy of Chinese Medicine, Changsha, Hunan, China; ^2^Department of Acupuncture, Xiangtan County Hospital of Traditional Chinese Medicine, Xiangtan, Hunan, China; ^3^Provincial Key Laboratory of TCM Diagnostics, Hunan University of Chinese Medicine, Changsha, Hunan, China; ^4^School of Traditional Chinese Medicine, Hunan University of Chinese Medicine, Changsha, Hunan, China; ^5^School of Integrated Chinese and Western Medicine, Hunan University of Chinese Medicine, Changsha, Hunan, China; ^6^Department of Scientific Research and Education, Hunan Provincial Hospital of Integrated Traditional Chinese and Western (The Affiliated Hospital of Hunan Academy of Traditional Chinese Medicine), Changsha, Hunan, China

**Keywords:** Cellular senescence, HMGA1, Liver hepatocellular carcinoma, Prognostic biomarker, Tumor microenvironment

## Abstract

**Background:**

High mobility group A1 (HMGA1) has emerged as a key oncogenic factor in various cancers, but its specific role in liver hepatocellular carcinoma (LIHC) remains incompletely understood. This study aimed to investigate the expression pattern, biological functions, immune associations, clinical relevance, and therapeutic potential of HMGA1 in LIHC.

**Methods:**

We conducted a multi-omics analysis integrating transcriptomic, proteomic, and clinical data from TCGA, CPTAC, and HPA databases. Functional enrichment, immune infiltration profiling, and survival analyses were performed. *In-vitro* assays, including CCK-8, colony formation, β-galactosidase staining, and wound healing, were used to validate HMGA1’s biological functions in LIHC cells.

**Results:**

HMGA1 was significantly overexpressed in LIHC at both mRNA and protein levels (*P*<0.001). High HMGA1 expression correlated with advanced pathological stage, metastasis, and elevated AFP levels (all *P*<0.001). Kaplan-Meier analysis revealed that elevated HMGA1 predicted poor overall survival (OS) (HR=1.83, 95% CI: 1.12–2.99, *P*=0.014), disease-specific survival (DSS) (HR=2.12, 95% CI: 1.33–3.35, *P*=0.002), and progression-free interval (PFI) (HR=1.68, 95% CI: 1.14–2.48, *P*=0.009). Multivariate Cox analysis confirmed HMGA1 as an independent prognostic factor for OS (HR=1.75, 95% CI: 1.11–2.76, *P*=0.016). A nomogram incorporating HMGA1 and clinicopathological variables showed good predictive performance with a 3-year AUC of 0.723. Functionally, HMGA1 knockdown suppressed LIHC cell proliferation (38.9% reduction in HepG2 and 46.0% in Huh-7 at 48h), migration (44–59% inhibition at 24h), and colony formation (41.8–44.2% reduction), while significantly inducing cellular senescence (3.4–3.5-fold increase in β-gal+cells, *P*<0.001). GSEA and immune analysis indicated that HMGA1 may promote immune evasion and senescence bypass.

**Conclusion:**

HMGA1 serves as a robust prognostic biomarker and functional driver of malignant progression in LIHC. Its integration into prognostic models may enhance risk stratification and guide personalized therapeutic strategies. Nevertheless, further *in-vivo* validation and prospective clinical studies are required to establish its translational applicability.

## Introduction

 Liver hepatocellular carcinoma (LIHC) is one of the most prevalent malignancies worldwide, ranking as the sixth most common cancer and the third leading cause of cancer-related death.^1^ Despite advances in surgical techniques, locoregional therapies, and systemic treatments such as targeted therapy and immunotherapy, the prognosis for patients with LIHC remains poor, with a 5-year survival rate below 20%.^2^ The high heterogeneity of LIHC and its frequent diagnosis at advanced stages underscore the urgent need for identifying novel biomarkers and therapeutic targets to improve patient outcomes.

 The high mobility group A1 (HMGA1), a non-histone chromatin structural protein, regulates tumor-related genes through pathways like Wnt/β-catenin and PI3K/Akt, influencing processes such as aging, apoptosis, and chemotherapy resistance, supporting its role as a candidate biomarker and chromatin remodeler rather than a therapeutic claim.^3^ Emerging evidence implicates HMGA1 as an oncogene in several malignancies, including breast, pancreatic, and colorectal cancers, where it promotes tumor growth, metastasis, and resistance to therapy.^4-6^ In LIHC specifically, HMGA1 is overexpressed and correlates with higher Edmondson grade and worse prognosis and independent datasets further suggest links to immune features.^7,8^ Nevertheless, how HMGA1 interfaces with senescence programs and the hepatic tumor microenvironment (TME) in LIHC remains incompletely defined.

 Recent studies suggest that tumor progression involves not only intrinsic oncogenic drivers but also extrinsic factors, such as the TME.^9^ The TME, comprising stromal cells, immune cells, and extracellular matrix components, plays a pivotal role in tumor growth and immune evasion.^10-12^ Additionally, cellular senescence, a state of irreversible cell cycle arrest, has emerged as a double-edged sword in cancer biology.^13^ While senescence suppresses tumorigenesis in early stages, the senescence-associated secretory phenotype (SASP) can promote tumor progression by remodeling the TME.^14^ Mechanistically, HMGA1 accumulates on senescent chromatin, is essential for senescence-associated heterochromatin foci (SAHF), and contributes to large-scale chromatin reorganization in senescent cells.^8,15^ In HCC models, HMGA1 activates an NF-κB-CCL2 axis that recruits macrophages and enhances tumor aggressiveness, providing a direct connection between HMGA1 and immune-inflammatory remodeling of the hepatic TME.^16^ Consistently, the CCL2/CCR2 pathway is a key route for monocyte/macrophage trafficking in HCC, and senescent hepatocytes deploy CCL2 as part of the SASP to orchestrate myeloid recruitment.^17,18^

 We hypothesized that HMGA1 is upregulated in LIHC and associates with adverse prognosis, and that HMGA1-related programs (cell-cycle/senescence and immune infiltration) contribute to disease progression. Our primary objective was to quantify HMGA1 expression across multi-omics datasets and evaluate its independent prognostic value in multivariable models; secondary objectives were to characterize HMGA1-linked biological pathways, immune infiltration patterns, and *in-vitro* phenotypes, and to explore whether adding HMGA1 to clinical covariates improves risk discrimination.

## Materials and Methods

###  Data Acquisition and Expression Analysis

 We analyzed the gene expression profile of HMGA1 in pan-cancer and their corresponding normal tissues using data from the The Cancer Genome Atlas (TCGA, https://portal.gdc.cancer.gov) and the Genotype-Tissue Expression (GTEx, https://gtexportal.org/home/) databases.^19,20^ With a particular focus on LIHC, we curated and analyzed RNA-seq data from both unpaired and paired samples available in the TCGA and GTEx databases. To ensure accurate normalization, standardization, and visualization, we employed the ‘limma’ package along with other R (v4.2.1) tools. Additionally, we performed a multi-omics analysis to examine the protein expression levels of HMGA1 in LIHC, leveraging the CPTAC data through the University of ALabama at Birmingham CANcer (UALCAN, https://ualcan.path.uab.edu) platform.^21^ The representative images of IHC staining of HMGA1 in LIHC paracancerous tissues and cancerous tissues were gained from the Human Protein Atlas (HPA, https://www.proteinatlas.org).^22^

###  Differential Expression Analysis of HMGA1

 TCGA-LIHC tumors were dichotomized into HMGA1-high and HMGA1-low by the cohort median of HMGA1 expression. Differential expression was performed in R v4.2.1 (Bioconductor v3.16) using DESeq2 v1.38.3. Multiple testing was controlled with Benjamini–Hochberg FDR via base stats v4.2.1; significance was defined as |log₂FC| > 1.5 and FDR < 0.05. Volcano plots were generated with ggplot2 v3.4.4.

 For co-expression, Spearman’s rank correlation between HMGA1 and all genes was computed across tumors using stats v4.2.1; *P* values were FDR-adjusted (Benjamini–Hochberg). The top 30 positively and top 30 negatively correlated genes (ranked by |ρ|, FDR < 0.05) were visualized as row-wise z-scored heatmaps using pheatmap v1.0.12.

 Significant DEGs were queried against STRING v12.0 (*Homo sapiens*; default evidence channels; medium confidence) and the network was rendered in Cytoscape v3.10.1 (layout by Prefuse Force Directed). Nodes represent proteins; edges represent STRING (https://string-db.org/) interactions.

###  Functional Enrichment Analysis

 To identify genes differentially expressed between HMGA1-high and HMGA1-low LIHC samples, differential expression analysis was performed using DESeq2 (version 1.40.1) in R (version 4.2.1). The thresholds for significance were set at absolute log2 fold change |log2FC| > 1 and Benjamini–Hochberg adjusted *P* value (FDR) < 0.05. Multiple testing correction was applied via the Benjamini–Hochberg procedure. Volcano plots were generated to visualize upregulated and downregulated genes.

 Spearman correlation analysis was conducted between HMGA1 expression and all other genes to identify co-expression patterns; correlation coefficients and corresponding p-values were computed, and multiple comparisons were corrected by FDR. The top positively and negatively correlated genes (e.g. top 30) were displayed in heatmaps to depict clustering between HMGA1-high and -low groups.

 The protein–protein interaction and network exploration of candidates were assisted by integrating known interactions using the STRING database (version 11.5) and visualized in Cytoscape (version 3.9.1) to help define hub gene relationships.

###  Immune Infiltration Analysis

 Immune infiltration was quantified by ssGSEA implemented in GSVA v1.46.0 on R v4.2.1 with Bioconductor v3.16 and GSEABase v1.58.0, using the 28-cell-type signatures of Bindea et al (as distributed via msigdbr v7.5.1). Tumors were dichotomized into HMGA1-high and HMGA1-low by the cohort median of HMGA1 expression. For group comparisons of ssGSEA scores, two-sided Wilcoxon rank-sum tests were applied and p values were adjusted across cell types with the Benjamini–Hochberg method (BH-FDR < 0.05 considered significant). Correlations between continuous HMGA1 expression and immune scores were assessed with Spearman’s ρ and BH correction across cell types. Pairwise correlations among immune scores were computed with Spearman’s p; the network was visualized using circlize v0.4.15 and ggplot2 v3.4.4. Unless otherwise stated, all multiple-testing adjustments used BH via base stats v4.2.1.

###  Clinical Statistical Analysis, Model Construction and Prognostic Evaluation

 Clinical baseline data for 424 LIHC patients were extracted from TCGA. Associations between HMGA1 expression and clinicopathological categorical variables (e.g. tumor status, AFP level stratified at 400 ng/mL) were assessed with Wilcoxon rank-sum tests for two-group comparisons and Kruskal-Wallis tests for multi-category comparisons (e.g. pathologic stage).HMGA1 was modeled both as a continuous variable (z-score of log2(TPM + 1)) and as a dichotomous variable (median split) in all downstream analyses, with the continuous specification considered primary.

 Prognostic analyses included Kaplan–Meier survival curves with log-rank tests, and both univariate and multivariable Cox proportional hazards regression models for overall survival (OS), disease-specific survival (DSS), and progression-free interval (PFI). Multivariable models adjusted *a priori* for T/N/M stage, pathologic stage, tumor status, AFP ( ≥ 400 vs < 400 ng/mL), age, gender, and height, subject to data availability and collinearity checks. The proportional hazards (PH) assumption was formally assessed using Schoenfeld residuals; where PH violations were detected, we pre-specified handling via (i) stratified Cox models for violating covariates and/or (ii) time-varying effects for HMGA1 (interaction with log-time). Hazard ratios (HRs) with 95% confidence intervals (CIs) and p-values were reported.

 A multivariable prognostic nomogram incorporating HMGA1 expression, T stage, M stage, pathologic stage, and tumor status was constructed based on the Cox model to predict 1-, 3-, and 5-year OS. Calibration of the nomogram was assessed via internal validation (bootstrap resampling with 1,000 iterations), comparing predicted vs observed outcomes. Model performance was summarized by the optimism-corrected concordance index (C-index) and time-dependent AUCs (timeROC).

###  Cell Culture

 HepG2 (CL-0103, Pricella Biotechnology Co., Ltd.) and Huh-7 (CL-0120, Pricella Biotechnology Co., Ltd.) cell lines were used to assess the functional impact of HMGA1 knockdown. Both cell lines were obtained from a reputable cell bank and maintained under sterile conditions. Cells were cultured in Dulbecco’s modified eagle medium (DMEM, PM150210, Pricella Biotechnology Co., Ltd.) supplemented with 10% fetal bovine serum (FBS, 164210, Pricella Biotechnology Co., Ltd.) and 1% penicillin-streptomycin (P/S, PB180120, Pricella Biotechnology Co., Ltd.) solution to prevent contamination. Cell cultures were incubated at 37°C in a humidified atmosphere containing 5% CO₂. For passaging, cells were washed with phosphate-buffered saline (PBS, PB180327, Pricella Biotechnology Co., Ltd.) and detached using 0.25% trypsin-EDTA solution. Subcultures were prepared every 2-3 days to maintain cell viability and ensure they remained in the exponential growth phase for transfection and subsequent assays.

###  Transfection Protocol HMGA1 Knockdown and Western Blot Analysis

 To investigate the functional role of HMGA1 in HCC, HepG2 and Huh-7 cell lines were employed. Cells were transfected with either scrambled siRNA (negative control) or one of three independent siRNAs (HMGA1-233, HMGA-1547, and HMGA1-1846) specifically targeting HMGA1 to reduce its expression.

###  Western Blotting Assay

 After transfection, cells were harvested, and protein extracts were prepared for WB analysis. WB was performed to confirm HMGA1(12094S, Cell Signaling Technology, Inc.) knockdown efficiency, with β-actin (4970S, Cell Signaling Technology, Inc.) used as a loading control. Protein bands corresponding to HMGA1 were visualized using enhanced chemiluminescence (ECL, 6883P3, Cell Signaling Technology, Inc.), and band intensities were quantified to assess the level of HMGA1 suppression in siRNA-treated cells compared to controls.

###  Cell Counting Kit-8 Assay

 To evaluate the impact of HMGA1 knockdown on cell viability, the CCK-8 assay was conducted on transfected HepG2 and Huh-7 cells. Following transfection with scrambled siRNA or HMGA1-targeting siRNAs, cells were seeded in 96-well plates at a density of 5,000 cells per well. After 24, 48 and 72 hours, 10 µL of CCK-8 solution (E-CK-A362, Elabscience Biotechnology Co., Ltd.) was added to each well and incubated at 37 °C for 2 hours. Absorbance was measured at 450 nm using a microplate reader, and cell viability was calculated relative to control wells.

###  Colony Formation Assay

 The colony formation assay was conducted to determine the effect of HMGA1 knockdown on cell proliferation. After transfection, HepG2 and Huh-7 cells were seeded in 6-well plates at a low density (500 cells per well) and cultured for 10-14 days to allow colonies to form. Cells were then fixed with 4% paraformaldehyde and stained with crystal violet (AWC0333, Abiowell Biotechnology Co., Ltd). Colonies (defined as clusters of > 50 cells) were counted under a microscope, and colony numbers in HMGA1 knockdown groups were compared to scrambled controls.

###  Senescence-Associated β-Galactosidase (SA-β-gal) Assay

 To assess cellular senescence induced by HMGA1 knockdown, a SA-β-gal staining kit (C0602, Beyotime Biotech, Co., Ltd) was used. HepG2 and Huh-7 cells transfected with either scrambled siRNA or HMGA1-targeting siRNAs were fixed with 2% paraformaldehyde and incubated with SA-β-gal staining solution at 37°C (pH 6.0) overnight. Senescent cells, indicated by blue staining, were observed under a light microscope, and percentages of β-gal-positive cells were calculated.

###  Wound-Healing Assay for Cell Migration

 The wound healing assay was used to examine the effect of HMGA1 knockdown on cell migration. Transfected HepG2 and Huh-7 cells were grown to 90% confluence in 6-well plates, and a sterile pipette tip was used to create a scratch (wound) across the cell monolayer. After washing with PBS to remove detached cells, fresh serum-free medium was added, and cells were incubated at 37°C. Images of the wound area were captured at 0 and 24 hours post-wounding using an inverted microscope.

###  Statistical Analysis

 All experiments were performed in triplicate. Data are presented as mean ± SD. Statistical significance was assessed using Student’s t-test or one-way ANOVA followed by Tukey’s post hoc test. *P* < 0.05 was considered statistically significant.

## Results

###  HMGA1 Expression in LIHC

**Figure 1:**
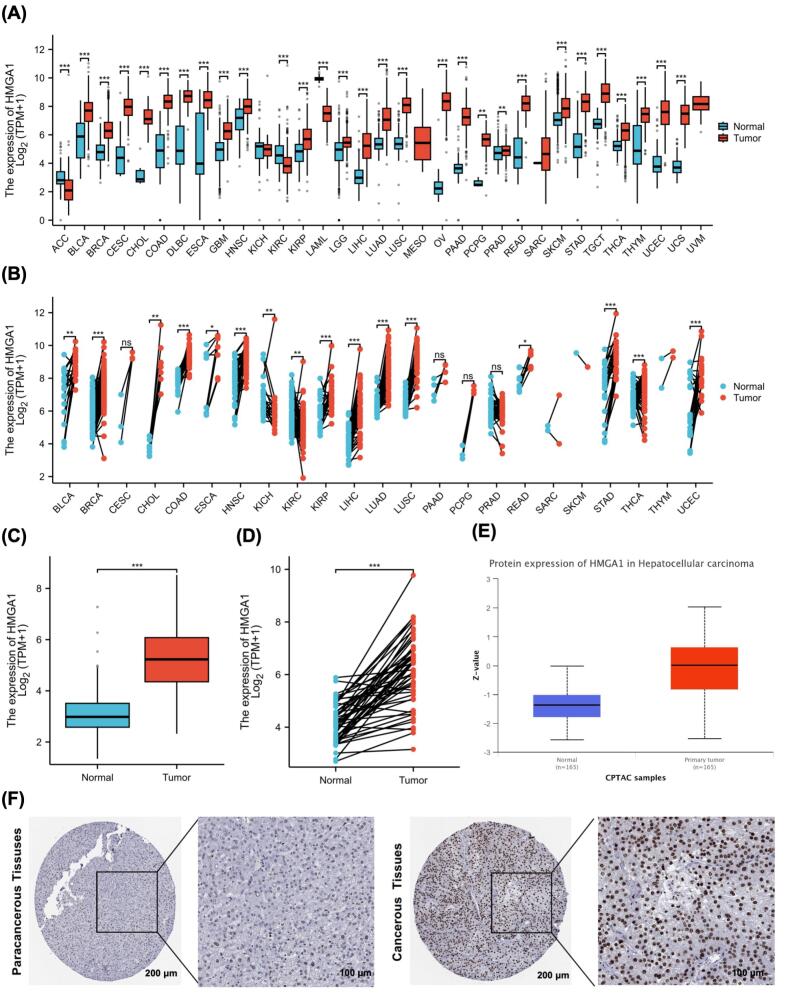
HMGA1 Expression Across Cancers and in LIHC. (A) Boxplots of HMGA1 mRNA levels (log₂[TPM + 1]) in unpaired tumor vs. normal tissues across 33 TCGA cancer types. (B) Paired tumor–adjacent normal comparisons across TCGA cancer types. Two-sided Wilcoxon signed-rank tests; lines connect matched samples from the same patient. Multiple-comparison correction across cancer types as in panel A. (C) TCGA-LIHC, unpaired tumor vs. normal HMGA1 mRNA (log₂[TPM + 1]). Two-sided Wilcoxon rank-sum test. (D) TCGA-LIHC, paired tumor–normal HMGA1 mRNA. Two-sided Wilcoxon signed-rank test; lines connect matched pairs. (E) HMGA1 protein abundance in LIHC from CPTAC (primary tumor vs. normal), displayed as Z-scores. Two-sided Wilcoxon rank-sum test. (F) Representative IHC images from the HPA showing HMGA1 staining in paracancerous and cancerous liver tissues.

 Across TCGA pan-cancer cohorts, HMGA1 transcript levels were broadly higher in tumor tissues than in matched normal controls (Figures 1A and 1B). Focusing on LIHC, both the unpaired comparison of tumors versus the normal (Figure 1C) and the paired analysis within the same individuals (Figure 1D) showed consistently elevated HMGA1 expression in tumors, with most paired samples exhibiting an upward shift from normal to tumor. At the protein level, CPTAC data similarly indicated higher HMGA1 abundance in LIHC tumors relative to normal tissues (Figure 1E). Immunohistochemistry from the Human Protein Atlas further corroborated these findings, revealing stronger nuclear staining of HMGA1 in hepatocellular carcinoma compared with paracancerous liver tissues (Figure 1F). Collectively, multi-omics and histological evidence support HMGA1 upregulation in LIHC.

###  Analysis of Single-gene Differential Expression of HMGA1 in LIHC

**Figure 2:**
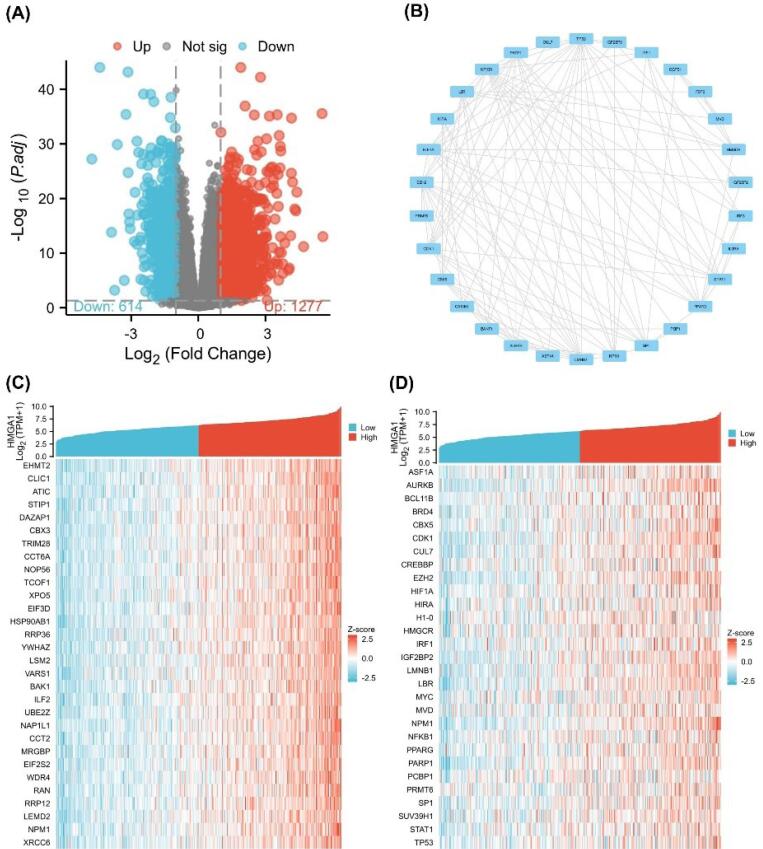
Differential Expression and Correlation Analysis of HMGA1 in LIHC. (A) Volcano plot of differentially expressed genes (DEGs) between HMGA1-high and HMGA1-low expression groups in LIHC. The x-axis represents log₂ (fold change), and the y-axis represents –log₁₀ (adjusted P-value). Red and blue dots indicate significantly upregulated (n = 1277) and downregulated (n = 614) genes, respectively, while gray dots represent non-significant genes (adjusted p ≥ 0.05). (B) PPI network of selected HMGA1-related DEGs constructed using the STRING database. Each node represents a protein, and edges represent known or predicted interactions. (C) Heatmap of the top 30 genes most positively or negatively correlated with HMGA1 expression in TCGA-LIHC samples, based on Spearman correlation. (D) Co-expression heatmap of 30 hub genes from the PPI network in HMGA1-high and HMGA1-low expression groups. Samples are grouped by HMGA1 expression status.

 Differential expression analysis between HMGA1-high and -low LIHC groups identified 1,277 upregulated and 514 downregulated genes (|log₂FC| > 1, adjusted p < 0.05; Figure 2A). A PPI network constructed from these DEGs comprised 1,150 nodes and 3,827 edges; the top 20 hub nodes—such as CDK1, CCNB1, and AURKB—were highlighted based on degree centrality (Figure 2B).

 Single-gene correlation analysis ranked genes by their association with HMGA1 expression. The top 30 positively correlated genes included E2F2, GINS2, MCM4, and CCNB2 (Figure 2C). A heatmap of these hub genes demonstrated distinct expression patterns between HMGA1-high and -low groups, with coordinated upregulation in the HMGA1-high cohort (Figure 2D).

###  Pathway Enrichment and Analysis of HMGA1 in LIHC

**Figure 3:**
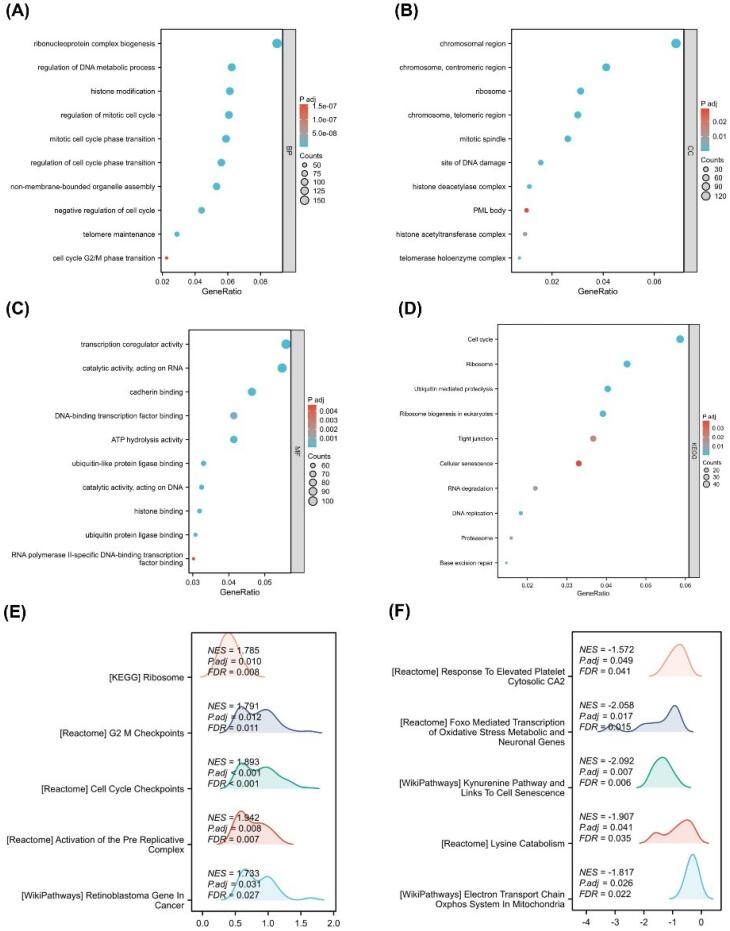
Functional Enrichment and Pathway Analysis of HMGA1-Associated Genes in LIHC. (A–C) GO enrichment dot plots for HMGA1-associated differentially expressed genes, shown for BP, CC, and MF. GeneRatio is plotted on the x-axis; dot size reflects the number of mapped genes (Counts); dot color encodes the adjusted p-value (Padj). (D) KEGG pathway enrichment for the same gene set; plotting conventions as in panels A–C. (E) GSEA for the HMGA1-high group. Representative positively enriched programs are displayed with the NES, Padj, and FDR as printed on the plots. (F) GSEA for the HMGA1-low group, highlighting negatively enriched programs with NES, Padj, and FDR as shown.

 To elucidate the biological roles of HMGA1-associated DEGs in LIHC, GO enrichment analysis was performed. In the BP category, DEGs were enriched in ribonucleoprotein complex biogenesis, regulation of DNA metabolic process, histone modification, regulation of mitotic cell cycle, mitotic cell cycle phase transition, and cell cycle G2/M phase transition (Figure 3A). CC terms included chromosomal region, centromeric region, telomeric region, and histone deacetylase complex (Figure 3B). MF enrichment highlighted activities such as transcription coregulator activity, catalytic activity acting on DNA, DNA-binding transcription factor binding, and ATP hydrolase activity (Figure 3C). KEGG analysis revealed significant enrichment in cell cycle, DNA replication, p53 signaling pathway, and base excision repair (Figure 3D).

 GSEA comparing HMGA1-high versus HMGA1-low groups showed that HMGA1-high samples were significantly enriched for pathways related to ribosome (NES = 1.785, FDR = 0.008), G2/M checkpoints (NES = 1.783, FDR = 0.015), cell cycle checkpoints (NES = 1.693, FDR = 0.040), activation of the pre-replicative complex (NES = 1.783, FDR = 0.022), and retinoblastoma gene regulation in cancer (NES = 1.683, FDR = 0.030) (Figure 3E). Conversely, HMGA1-low samples exhibited enrichment in pathways such as response to elevated platelet cytosolic Ca^2+^ (NES = –1.572, FDR = 0.046), exoribonuclease-mediated transcription (NES = –2.050, FDR = 0.006), lysine catabolism (NES = –1.907, FDR = 0.033), and electron transport chain in mitochondria (NES = –1.817, FDR = 0.022) (Figure 3F).

###  Relationship Between Immune Infiltration and HMGA1 Expression

**Figure 4:**
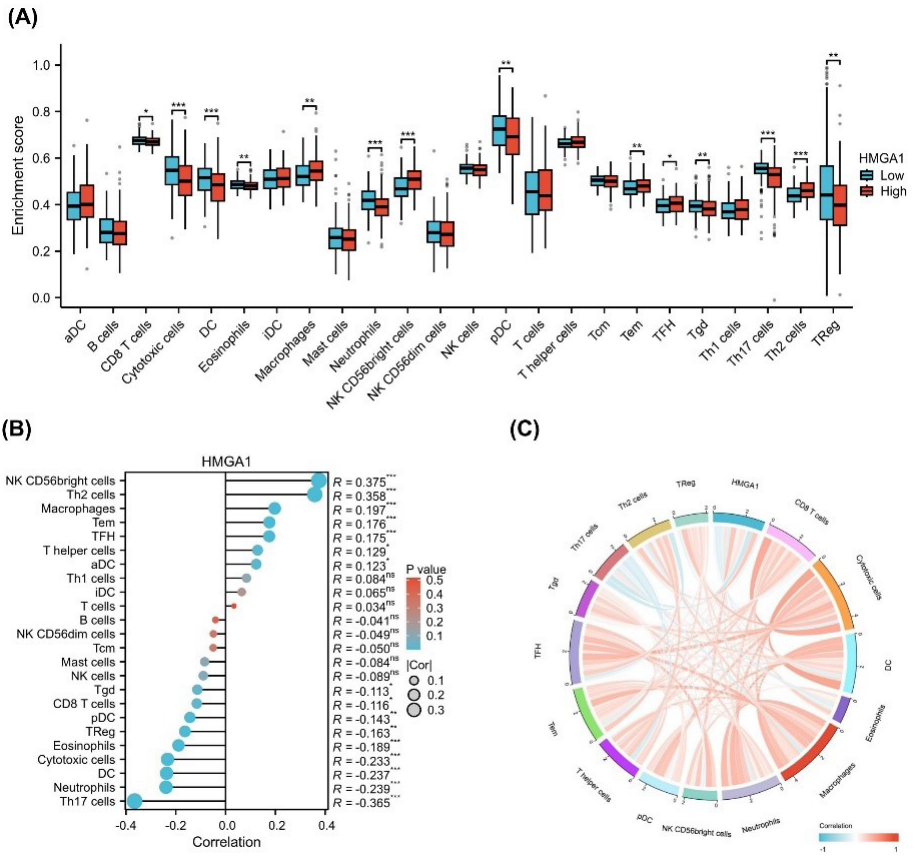
Immune Infiltration Analysis of HMGA1. (A) Boxplot showing differences in immune cell infiltration scores (ssGSEA) between HMGA1-high and HMGA1-low expression groups in LIHC. Immune cell types include T cells, B cells, NK cells, macrophages, and others. (B) Spearman correlation between HMGA1 expression and infiltration scores of 24 immune cell types. Each point represents a cell type; blue and red colors indicate negative and positive correlations, respectively. Statistical significance is denoted by asterisks (^*^P < 0.05, ^**^ P < 0.01, ^***^ P < 0.001). (C) Circular chord diagram displaying the correlation network between HMGA1 and selected immune cell types, colored by the direction and strength of correlation.

 To explore the immunological role of HMGA1 in LIHC, we assessed the infiltration levels of 24 immune cell types by comparing HMGA1-high and HMGA1-low expression groups. As shown in Figure 4A, a significant alteration in the immune landscape was observed between the two groups. Specifically, HMGA1-high samples exhibited increased enrichment scores for several immune subsets, including macrophages, NK CD56^bright^ cells, Tem, TFH cells, and Th2 cells (*P* < 0.001). In contrast, the infiltration of CD8 T cells, cytotoxic cells, DCs, eosinophils, neutrophils, pDCs, Tgd, Th17 cells, and Tregs was significantly reduced in the HMGA1-high group (*P* < 0.001).

 To validate these observations, a correlation analysis was performed (Figure 4B). The results showed that HMGA1 expression was positively correlated with the infiltration of NK CD56^bright^ cells (R = 0.375), Th2 cells (R = 0.358), macrophages (R = 0.197), and TFH cells (R = 0.176). Conversely, negative correlations were observed with Th17 cells (R = –0.365), neutrophils (R = –0.239), and cytotoxic cells (R = –0.233) (all *P* < 0.05).

 To further visualize the global immune correlation network, a chord diagram was generated (Figure 4C). The diagram illustrates the spectrum of associations between HMGA1 and various immune cell types, with red lines representing positive correlations and blue lines indicating negative associations. Notably, HMGA1 demonstrated strong positive connectivity with immunoregulatory or immunosuppressive cell types, including Th2 cells, Tregs, macrophages, and NK CD56bright cells, suggesting a potential role in fostering an immunosuppressive microenvironment. In contrast, negative associations were observed with key effector immune cells such as cytotoxic cells, Th17 cells, and neutrophils, which are typically involved in anti-tumor responses.

 To further support these findings, scatter plots depicting the relationship between HMGA1 expression and immune cell infiltration scores were generated, with statistically significant correlations (*P* < 0.001) presented in Figure S1 (Supplementary file 1).

###  Association of HMGA1 Expression with Clinicopathological Features and Prognosis in LIHC

**Figure 5:**
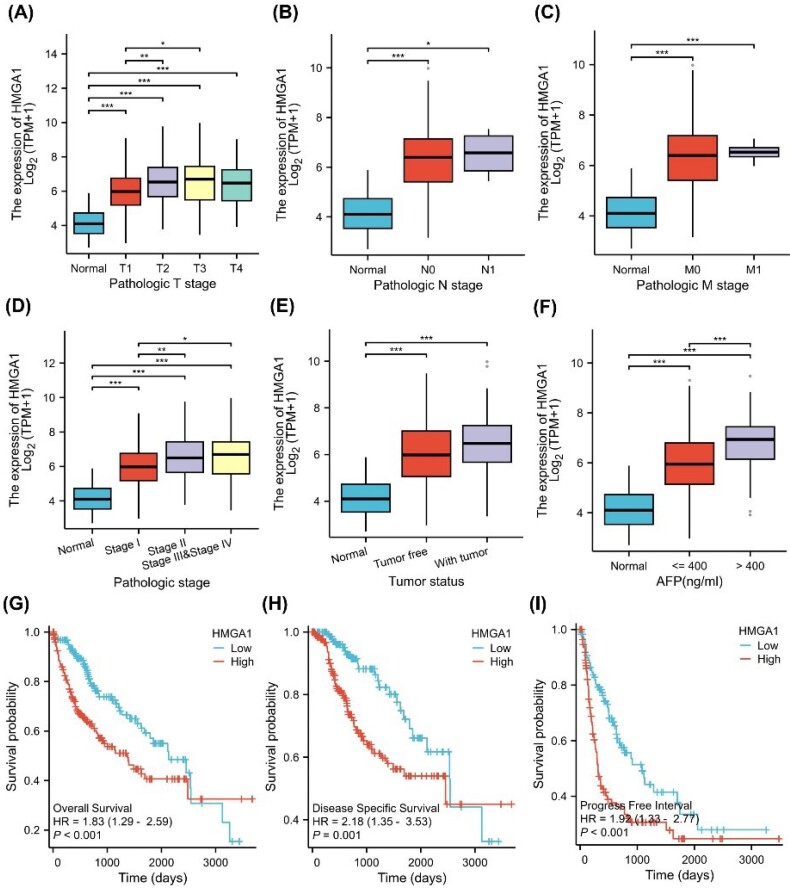
Clinical Relevance and Prognostic Value of HMGA1 Expression in LIHC. (A–F) Associations between HMGA1 mRNA levels (log₂[TPM + 1]) and clinicopathologic variables in TCGA-LIHC: (A) Pathologic T stage, (B) N stage, (C) M stage, (D) overall pathologic stage (I–IV), (E) tumor status (normal, tumor-free, with tumor), and (F) AFP groups (normal, ≤ 400 ng/mL, > 400 ng/mL). (G–I) Kaplan–Meier analyses comparing HMGA1-high vs HMGA1 low tumors (median split) for OS, DSS, and PFI, respectively. Curves were compared with two-sided log-rank tests. HRs and 95% Cis shown on the panels derive from univariable Cox models.

 To investigate the correlation between HMGA1 expression and clinicopathological characteristics, we utilized clinical baseline data from 424 LIHC samples obtained from the TCGA database (Table 1). As shown in Figure 5A-F, the correlation between HMGA1 expression and clinical data across different pathological stages was consistent with the clinical baseline findings. Specifically, HMGA1 expression significantly increased with advanced pathologic T stage, with the highest levels observed in T3 and T4 stages compared to normal tissue (*P* < 0.001) (Figure 5A). A similar trend was observed for the pathologic N stage, where N1 patients showed significantly higher HMGA1 expression than normal controls (*P* < 0.001) (Figure 5B). For the pathologic M stage, patients with metastasis (M1) displayed elevated HMGA1 expression relative to normal and non-metastatic (M0) groups (*P* < 0.001) (Figure 5C).

**Table 1:** Clinical Baseline Information about the Association between the Expression of HMGA1 and Different Clinical–Pathological Characteristics of LIHC Patients from the TCGA Database

**Characteristics**	**Low expression of HMGA1** **(n=187)**	**High expression of HMGA1** **(n=187)**	***P*** ** value**
Pathologic T stage, n (%)			
T1	110 (29.6%)	73 (19.7%)	< 0.001
T2	36 (9.7%)	59 (15.9%)	
T3	32 (8.6%)	48 (12.9%)	
T4	6 (1.6%)	7 (1.9%)	
Pathologic stage, n (%)			
Stage I	102 (29.1%)	71 (20.3%)	0.004
Stage II	35 (10%)	52 (14.9%)	
Stage III	33 (9.4%)	52 (14.9%)	
Stage IV	2 (0.6%)	3 (0.9%)	
Tumor status, n (%)			
Tumor-free	113 (31.8%)	89 (25.1%)	0.032
With tumor	68 (19.2%)	85 (23.9%)	
Race, n (%)			
Asian	68 (18.8%)	92 (25.4%)	0.018
Black or African American	7 (1.9%)	10 (2.8%)	
White	106 (29.3%)	79 (21.8%)	
Weight, n (%)			
< = 70	81 (23.4%)	103 (29.8%)	0.009
> 70	94 (27.2%)	68 (19.7%)	
Histological type, n (%)			
Fibrolamellar carcinoma	3 (0.8%)	0 (0%)	0.037
Hepatocellular carcinoma	183 (48.9%)	181 (48.4%)	
Hepatocholangiocarcinoma (mixed)	1 (0.3%)	6 (1.6%)	
Residual tumor, n (%)			
R0	171 (49.6%)	156 (45.2%)	0.018
R1	4 (1.2%)	13 (3.8%)	
R2	0 (0%)	1 (0.3%)	
Histologic grade, n (%)			
G1	35 (9.5%)	20 (5.4%)	< 0.001
G2	106 (28.7%)	72 (19.5%)	
G3	40 (10.8%)	84 (22.8%)	
G4	3 (0.8%)	9 (2.4%)	
AFP (ng/ml), n (%)			
≤ 400	126 (45%)	89 (31.8%)	< 0.001
> 400	18 (6.4%)	47 (16.8%)	
OS event, n (%)			
Alive	134 (35.8%)	110 (29.4%)	0.009
Dead	53 (14.2%)	77 (20.6%)	

 When stratified by pathologic stage, HMGA1 expression progressively increased from early (stage I) to advanced stages (stage III and IV), with significant differences between stages (*P* < 0.05) (Figure 5D). Tumor status analysis revealed that HMGA1 expression was significantly higher in tumor tissues compared to non-tumor tissues (*P* < 0.001) (Figure 5E). Additionally, HMGA1 expression was elevated in patients with serum AFP levels > 400 ng/mL compared to those with levels ≤ 400 ng/mL (*P* < 0.001) (Figure 5F).

 The prognostic value of HMGA1 in LIHC was assessed using Kaplan-Meier survival curves (Figure 5G-I). High HMGA1 expression was associated with worse OS (*HR*= 1.83, 95% CI: 1.12–2.99, *P <*0.05) (Figure 5G), DSS (*HR*= 2.12, 95% CI: 1.33–3.35, *P <*0.01) (Figure 5H), and PFI (*HR*= 1.68, 95% CI: 1.14–2.48, *P <*0.01) (Figure 5I). Figure S2 shows the survival analysis results for various subgroups, revealing that higher HMGA1 expression is associated with poorer survival outcomes.

###  Prognostic Value of HMGA1 and Construction of a Survival Nomogram in LIHC

**Figure 6:**
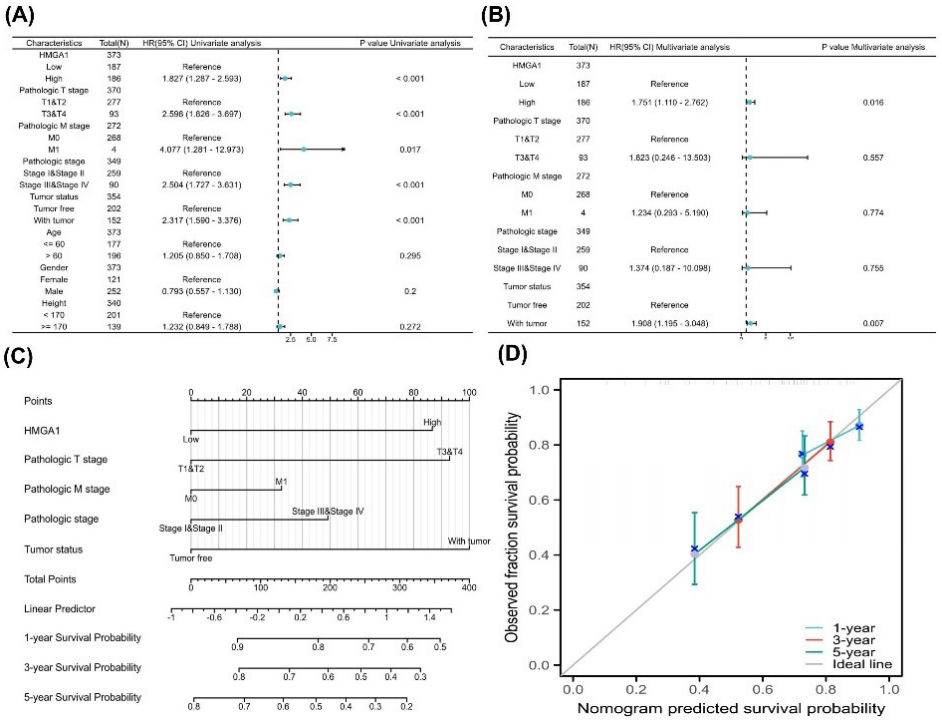
Prognostic Modeling of HMGA1 in LIHC. (A) Univariable Cox analyses for overall survival (OS) in TCGA-LIHC (N ≈ 373). Forest plot shows HR (95% CI) and two-sided p-values for HMGA1 (high vs low; median split of log₂[TPM + 1]) and available clinicopathologic covariates (e.g., pathologic T/N/M, overall pathologic stage, tumor status, age, gender; height; reference levels indicated on the plot). (B) Multivariable Cox model including HMGA1 and the covariates listed in panel A. Forest plot reports adjusted HRs (95% CI) and two-sided p-values; reference categories are shown on the left. (C) PH diagnostics for the multivariable model using Schoenfeld residuals. Each panel displays the smoothed residual trend (solid line) with 95% bands. (D) Nomogram predicting 1-, 3-, and 5-year OS based on the multivariable Cox model (constructed with the rms package). Points assigned to each predictor sum to a total score, which maps to predicted survival probabilities via the model’s baseline survival. (E) Calibration curves for the nomogram at 1, 3, and 5 years. The diagonal grey line represents perfect calibration. Colored curves show the bias-corrected fit; points represent grouped predictions with 95% CIs as vertical bars.

 To determine the prognostic significance of HMGA1 in LIHC, univariate and multivariate Cox regression analyses were performed for OS. As shown in Figure 6A, univariate analysis indicated that high HMGA1 expression (HR = 1.83, 95% CI: 1.287–2.593, *P*< 0.001), along with advanced T stage, M stage, pathologic stage, and tumor presence, were significantly associated with poor OS. In the multivariate analysis (Figure 6B), HMGA1 remained an independent prognostic factor (HR = 1.75, 95% CI: 1.110–2.762, *P* = 0.016), together with tumor status (HR = 1.91, *P* = 0.007).

 A prognostic nomogram incorporating HMGA1 expression, T stage, M stage, pathologic stage, and tumor status was constructed to predict 1-, 3-, and 5-year OS probabilities (Figure 6C). The calibration plot (Figure 6D) demonstrated excellent agreement between predicted and observed survival outcomes across all three time points, indicating the model’s good predictive performance.

###  Knockdown Efficiency of HMGA1

**Figure 7:**
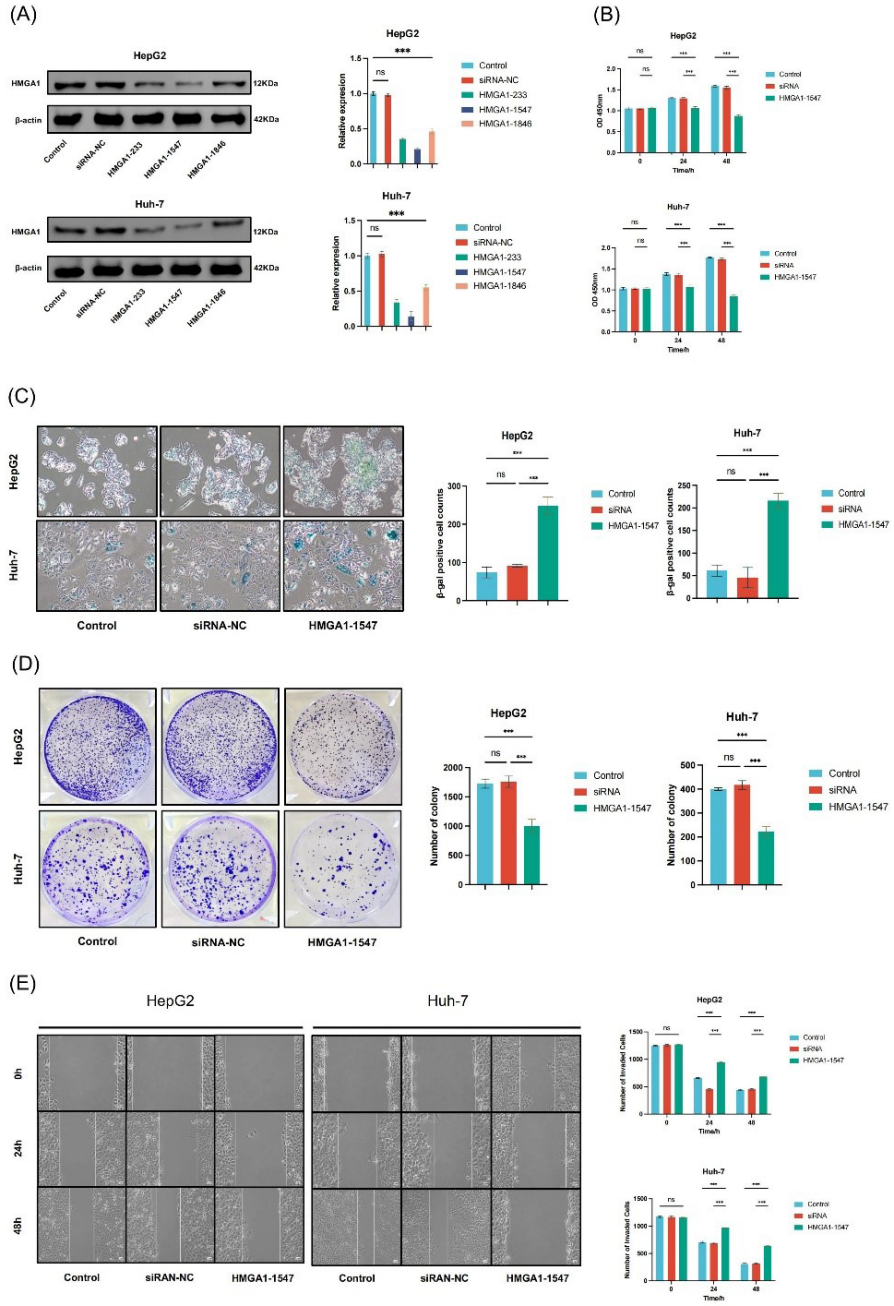
HMGA1 Silencing Induces Senescence and Suppresses Viability, Clonogenic Growth, and Migration in LIHC Cells. (A) Western-blot validation of HMGA1 silencing in HepG2 and Huh-7. β-actin serves as loading control. Right: densitometric quantification relative to control/siRNA-NC as plotted. (B) Short-term cell viability (CCK-8, 0/24/48 h) in HepG2 and Huh-7 for Control, siRNA-NC, and HMGA1-1547 groups (values as shown in the bar charts). (C) SA-β-gal staining images and quantification (% β-gal–positive cells) after HMGA1 knockdown versus controls. (D) Colony-formation assay: representative plates and quantified colony numbers per well for the indicated groups. (E) Wound-healing (scratch) migration assay at 0/24/48 h in HepG2 and Huh-7; right panels show the corresponding quantification as displayed.

 To evaluate the knockdown efficiency of HMGA1, HepG2 and Huh-7 cells were transfected with three specific siRNAs (siRNA-HMGA1-233, -1547, and -1846) and a negative control (siRNA-NC). Western blot analysis confirmed that all three siRNAs reduced HMGA1 protein levels to varying degrees, with siRNA-HMGA1-1547 exhibiting the most pronounced silencing effect in both cell lines (*P* < 0.001). Densitometric quantification further validated the knockdown efficiency of siRNA-HMGA1-1547 (Figure 7A). The original blots are provided in Figure S3 (Supplementary file 1).

###  Impact of HMGA1 Knockdown on Cell Proliferation

 Cell viability was assessed using the CCK-8 assay. In HepG2 cells, HMGA1-1547 reduced cell viability by approximately 15.9% at 24 hours (OD: 1.26 ± 0.03 vs 1.50 ± 0.02 in control, n = 3, *P* < 0.01) and 38.9% at 48 hours (OD: 1.11 ± 0.04 vs 1.82 ± 0.02, n = 3, *P* < 0.001). In Huh-7 cells, viability decreased by 19.2% at 24 hours (OD: 1.28 ± 0.02 vs 1.58 ± 0.02, n = 3, *P* < 0.01) and 46.0% at 48 hours (OD: 1.08 ± 0.03 vs 1.99 ± 0.03, n = 3, *P* < 0.001). HMGA1-1547 significantly suppressed cell proliferation at both 24 and 48 hours in HepG2 and Huh-7 cells compared to the control and siRNA-NC groups (*P* < 0.001, Figure 7B). No significant difference was observed between the control and siRNA-NC groups (*P* > 0.05), supporting the specificity of the knockdown.

###  Induction of Cellular Senescence

 Cellular senescence was evaluated by β-galactosidase staining. In HepG2 cells, the mean number of β-gal-positive senescent cells increased from 73.7 ± 12.9 in the control group and 91.0 ± 3.6 in the siRNA-NC group to 248.3 ± 23.0 in the HMGA1-1547 group (n = 3, *P* < 0.001). In Huh-7 cells, senescent cells increased from 61.3 ± 12.9 in the control group and 45.7 ± 18.0 in the siRNA-NC group to 215.3 ± 16.2 in the HMGA1-1547 group (n = 3, *P* < 0.001). Cells transfected with siRNA-HMGA1-1547 showed a marked increase in β-gal-positive cells, indicating enhanced senescence induction following HMGA1 depletion. This corresponds to approximately a 3.4-fold increase in HepG2 and a 3.5-fold increase in Huh-7 cells compared to their respective controls. This effect was consistently observed in both cell lines (Figure 7C).

###  Colony Formation Assay

 To assess clonogenic capacity, a colony formation assay was performed. In HepG2 cells, the mean number of colonies was 1726.7 ± 65.3 in the control group, and 1758.3 ± 96.2 in siRNA-NC which significantly decreased to 1005.0 ± 110.3 in the HMGA1-1547 group (n = 3, *P* < 0.001), corresponding to a 41.8% reduction compared to control. In Huh-7 cells, colony numbers were 399.7 ± 4.6 in control, 417.0 ± 18.6 in siRNA-NC, and 223.0 ± 20.2 in HMGA1-1547-treated cells (n = 3, *P* < 0.001), representing a 44.2% reduction compared to control. HMGA1 knockdown significantly reduced the number of colonies formed compared to the control and siRNA-NC groups in both HepG2 and Huh-7 cells, indicating impaired clonogenic potential following HMGA1 depletion (Figure 7D).

###  Effect of HMGA1 Knockdown on Cell Migration

 A wound-healing assay was performed to evaluate the effect of HMGA1 silencing on LIHC cell migration. In HepG2 cells, the mean wound closure (change in width) at 24 hours was 589.7 ± 14.5 μm in the control group, 585.8 ± 12.9 μm in siRNA-NC, and 327.8 ± 8.7 μm in the HMGA1-1547 group (n = 3, *P* < 0.001). At 48 hours, closure reached 805.9 ± 19.6 μm in control, 804.1 ± 21.3 μm in siRNA-NC, and 586.2 ± 15.2 μm in HMGA1-1547 cells (n = 3, *P* < 0.01), corresponding to an inhibition of approximately 44% at 24 hours and 27% at 48 hours. In Huh-7 cells, wound closure at 24 hours was 462.3 ± 12.3 μm in control, 476.7 ± 15.4 μm in siRNA-NC, and 187.5 ± 5.1 μm in HMGA1-1547 cells (n = 3, *P* < 0.001). At 48 hours, closure was 859.9 ± 21.4 μm in control, 848.7 ± 18.5 μm in siRNA-NC, and 521.5 ± 9.8 μm in HMGA1-1547 cells (n = 3, *P* < 0.001), representing an inhibition of approximately 59% at 24 hand 39% at 48 hours. Overall, HMGA1 knockdown significantly delayed wound closure in both LIHC cell lines, indicating impaired migratory capacity. No significant difference was observed between the control and siRNA-NC groups (*P* > 0.05), confirming the specificity of the knockdown effect (Figure 7E).

 Collectively, these results demonstrate that HMGA1 plays a crucial role in maintaining the proliferative, migratory, and clonogenic potential of LIHC cells while suppressing cellular senescence. Targeted silencing of HMGA1 significantly impairs these malignant phenotypes, highlighting its potential as a therapeutic target in hepatocellular carcinoma.

## Discussion

 Our study integrates multi-omics profiling, immune deconvolution, clinical association testing, survival modeling, and *in-vitro* perturbations to characterize the role of HMGA1 in LIHC. We confirmed marked overexpression at the mRNA and protein levels, and identified 1,277 upregulated and 614 downregulated genes in HMGA1-high tumors, indicating broad transcriptional reprogramming. Functionally coherent enrichments in cell-cycle regulation, DNA replication, chromatin remodeling, and mitotic control align with HMGA1’s canonical role as an architectural transcription factor that facilitates proliferative programs. Together with our cell-based assays (Figure 7A–E) showing efficient HMGA1 knockdown by Western blot, reduced short-term viability (CCK-8, 24–48 hours), decreased clonogenic survival, delayed wound closure (migration), and increased SA-β-gal positivity, these findings support a model in which HMGA1 sustains malignant growth partly by bypassing senescence checkpoints and maintaining cell-cycle transit and motility programs.^18,23,24^

 The PPI network constructed from these DEGs revealed 30 hub genes significantly correlated with HMGA1 expression, including canonical regulators of tumor progression such as TP53, EZH2, NFKB1, and HIF1A. These genes are centrally involved in chromatin remodeling, cell cycle control, immune modulation, and metabolic reprogramming—hallmarks of cancer.^15,25-27^ Their strong co-expression with HMGA1, visualized in heat maps and correlation analyses, supports the hypothesis that HMGA1 may serve as a key upstream modulator orchestrating a pro-tumorigenic gene expression program.

 Mechanistically, recent work demonstrates that HMGA1 reshapes higher-order chromatin compartments relevant to the senescence program, offering a structural basis for the broad transcriptional effects we observe.^28,29^ Moreover, literature links HMGA1 to p53 pathway control, EZH2-mediated epigenetic repression, and NF-κB inflammatory signaling, which provides convergent routes to cell-cycle activation and immune evasion in liver cancer.^16,30,31^

 Of note, hub genes such as EZH2 and NFKB1 provide insight into HMGA1’s role in shaping both intrinsic oncogenic signaling and the extrinsic TME. EZH2, a histone methyltransferase, is known for promoting epigenetic silencing and stemness, while NFKB1 is critical for inflammatory signaling and immune escape.^32,33^ Their integration into the HMGA1 regulatory axis highlights a plausible mechanism through which HMGA1 may contribute to both tumor growth and immune tolerance. Their integration into the HMGA1 regulatory axis highlights a plausible mechanism through which HMGA1 may contribute to both tumor growth and immune tolerance; experimental data in HCC showing HMGA1-driven NF-κB recruitment and CCL2 induction directly supports this axis.^31^

 Furthermore, the inclusion of genes related to cholesterol/mevalonate biosynthesis (*HMGCR*, *FDPS*, *GGPS1*) and immune-stress response (*STAT1*, *IRF1*, *IRF3*) within the HMGA1-correlated network suggests that HMGA1 may impact metabolic reprogramming and immune surveillance.^34-39^ Independent functional and genetic studies underscore the oncogenic dependence of liver and other cancers on the mevalonate program and its upstream stress regulators, offering a mechanistic bridge between cell-cycle/tumor suppression and lipid metabolism.^40-42^ These findings position HMGA1 not only as a transcriptional modulator but also as a nexus for metabolic-immune crosstalk in LIHC progression.

 To better understand the molecular processes associated with HMGA1 in tumor growth, functional enrichment analyses were performed. These results revealed that HMGA1-related genes are involved in crucial BP, including DNA metabolic process regulation, histone modification, and mitotic cell cycle transitions, all of which are closely tied to cellular senescence mechanisms.^43-45^ Specifically, the regulation of mitotic cell cycle phase transitions and cell cycle phase progression is essential in maintaining the balance between normal cellular function and the onset of senescence.^45,46^ Dysregulation of these processes, as influenced by HMGA1, may predispose cells to bypass senescence, fostering uncontrolled proliferation and tumor progression. This interpretation is coherent with high-resolution studies showing HMGA1’s role in senescence-associated chromatin and classical reports that HMGA proteins accumulate on the chromatin of senescent cells and help rewire the transcriptional landscape.^29^

 Importantly, the phenotypic constellation we observed after HMGA1 silencing—loss of viability and clonogenicity together with impaired migration and induction of SA-β-gal—provides an experimental bridge from our transcriptomic signals (G2/M and DNA-replication terms) to a cellular state consistent with checkpoint activation and senescence entry.^47^ The concurrent attenuation of migration further suggests that HMGA1 contributes to EMT/motility-linked programs, which is compatible with EZH2 and inflammatory (NF-κB/CCL2) axes implicated in HCC aggressiveness.^30,31^ While our assays were performed in 2D culture, the directionality of effects was concordant in HepG2 and Huh-7, arguing against cell-line idiosyncrasy; future rescue experiments (re-expression of HMGA1), orthogonal senescence markers (p16^INK4a^/p21^CIP1^) and *in-vivo* validation will be important to establish causality and generalizability.

 GSEA analysis identified significant enrichment of pathways such as the G2/M checkpoint and cell cycle checkpoints, processes that are central to the onset of cellular senescence.^48^ The disruption of these checkpoints by HMGA1 could allow cells to evade senescence, thereby sustaining proliferation despite genomic instability. Additionally, we observed enrichments related to the kynurenine pathway, a major immune-metabolic axis in which IDO1/TDO2-derived kynurenine activates AhR to promote regulatory programs and suppress effector immunity; mounting evidence links this axis to SASP modulation and tumor-promoting inflammation, offering a plausible route by which HMGA1-high tumors might sculpt a senescence-associated, immunosuppressive microenvironment.^49^

 The immune infiltration analysis further uncovered a shift toward immunoregulatory phenotypes in HMGA1-high tumors, with relative increases in macrophages, NK CD56^bright^, Th2, and TFH signatures and reductions in cytotoxic and Th17-associated compartments. This pattern is congruent with authoritative HCC literature describing NK dysfunction (skewing from cytotoxic CD56^dim^ to CD56^bright^ subsets) and accumulation of regulatory populations (e.g. Tregs/MDSCs) during tumor progression.^50,51^ Regarding Th lineages, intratumoral IL-17/Th17 has frequently been associated with worse outcomes and STAT3-driven protumor signaling in HCC, although context-dependent roles and plasticity have been reported across cancers.^52-54^ Collectively, these data situate our HMGA1-linked immune landscape within a broader framework of immune escape and checkpoint-refractory TME reprogramming in LIHC.

 The correlation between HMGA1 expression and clinicopathological characteristics reinforces its role as a biomarker of disease progression in LIHC. Elevated HMGA1 expression was associated with advanced pathological stages, metastatic disease, and higher serum AFP levels, suggesting its involvement in LIHC aggressiveness. The gradual increase in HMGA1 expression from early to advanced stages, coupled with its upregulation in metastatic and AFP-high groups, indicates its role in tumor proliferation and dissemination. These findings align with previous studies implicating HMGA1 in driving oncogenic transcriptional programs and promoting epithelial-mesenchymal transition, which facilitates metastasis.

 The prognostic analysis provides robust evidence of HMGA1 as an independent predictor of poor survival outcomes in LIHC. High HMGA1 expression was significantly associated with reduced OS, DSS, and PFI. Importantly, multivariate Cox regression analysis confirmed that HMGA1 expression independently predicts OS, reinforcing its clinical utility as a prognostic biomarker. The integration of HMGA1 into a nomogram alongside other clinicopathological factors, such as pathological stage and AFP levels, further emphasizes its potential for personalized risk stratification in LIHC patients. The high concordance between predicted and observed survival probabilities validates the model’s predictive performance.

 Collectively, our findings establish HMGA1 as a multifaceted driver of LIHC progression through transcriptional regulation, immune modulation, and promotion of tumor cell viability. Its robust association with poor prognosis and key pathological features supports its candidacy as both a prognostic biomarker and a potential therapeutic target in LIHC.

 While our study presents compelling evidence for the oncogenic role of HMGA1 in LIHC, there are several important limitations to consider. First, the use of publicly available datasets for the transcriptomic analysis introduces potential biases due to variations in data quality, patient populations, and technical methods. These datasets may not fully represent the heterogeneity of the disease, particularly in different ethnic groups or clinical settings. Therefore, the findings need to be validated in independent patient cohorts to confirm their generalizability.

 Second, reliance on *in-vitro* models for functional experiments, such as HMGA1 knockdown in HepG2 and Huh-7 cells, presents another limitation. While these cell lines are widely used in cancer research, they may not fully recapitulate the complexity of LIHC *in vivo*, including tumor heterogeneity, immune interactions, and stromal contributions. These models also lack the full range of tumor microenvironmental factors, which could influence HMGA1’s role in tumor progression. To further validate our findings, *in-vivo* models—such as xenograft models or genetically engineered mouse models—are essential to study the effects of HMGA1 inhibition on tumor growth, metastasis, and immune modulation in a more physiologically relevant context.

 Finally, while the immune infiltration analysis suggests a role for HMGA1 in modulating immune responses, it is based on correlative data. The functional impact of HMGA1 on immune cells in the TME remains to be fully elucidated. Future studies using *in-vivo* models and clinical samples will be crucial to validate these findings and further explore the immunomodulatory effects of HMGA1 in LIHC.

## Conclusion

 This study delineates a consistent role of HMGA1 in LIHC across transcriptomic/proteomic profiling, clinicopathologic association, survival modeling, and *in-vitro* perturbation. HMGA1 is overexpressed in tumors, aligns with adverse tumor biology, and—after adjustment for established covariates—retains prognostic value for survival. Mechanistically oriented analyses converge on cell-cycle/DNA-replication programs and immune-metabolic circuits, and functional assays show that HMGA1 silencing attenuates viability, clonogenic growth, and migration while promoting senescence, reinforcing biological plausibility.

 From a translational perspective, HMGA1 is best positioned at present as a prognostic biomarker that may augment individualized risk stratification when combined with standard factors. Given potential time-varying effects observed in diagnostics, future validation should incorporate REMARK-aligned external cohorts, calibration/decision-curve analyses, and time-dependent Cox or landmarking to refine clinical interpretability across LIHC etiologies.

 Therapeutic implications remain hypothesis-generating. Priority next steps include *in-vivo* loss-of-function studies, genetic/pharmacologic rescue, and mechanistic dissection of HMGA1’s links to senescence/SASP, mevalonate–cholesterol, and kynurenine–AhR axes, including combination strategies. Collectively, our findings nominate HMGA1 as a robust prognostic marker and a plausible therapeutic candidate that warrants rigorous mechanistic and preclinical validation before clinical translation.

## Supplementary Files

Supplementary file 1 contains Figures S1-S3.

## References

[1] Bray F, Laversanne M, Sung H, Ferlay J, Siegel RL, Soerjomataram I (2024). Global cancer statistics 2022: GLOBOCAN estimates of incidence and mortality worldwide for 36 cancers in 185 countries. CA Cancer J Clin.

[2] Jiang Y, Han QJ, Zhang J (2019). Hepatocellular carcinoma: mechanisms of progression and immunotherapy. World J Gastroenterol.

[3] Wang L, Zhang J, Xia M, Liu C, Zu X, Zhong J (2022). High mobility group A1 (HMGA1): structure, biological function, and therapeutic potential. Int J Biol Sci.

[4] Zhao Y, Liu MJ, Zhang L, Yang Q, Sun QH, Guo JR (2024). High mobility group A1 (HMGA1) promotes the tumorigenesis of colorectal cancer by increasing lipid synthesis. Nat Commun.

[5] Senigagliesi B, Penzo C, Severino LU, Maraspini R, Petrosino S, Morales-Navarrete H (2019). The high mobility group A1 (HMGA1) chromatin architectural factor modulates nuclear stiffness in breast cancer cells. Int J Mol Sci.

[6] Chia L, Wang B, Kim JH, Luo LZ, Shuai S, Herrera I (2023). HMGA1 induces FGF19 to drive pancreatic carcinogenesis and stroma formation. J Clin Invest.

[7] Andreozzi M, Quintavalle C, Benz D, Quagliata L, Matter M, Calabrese D (2016). HMGA1 expression in human hepatocellular carcinoma correlates with poor prognosis and promotes tumor growth and migration in in vitro models. Neoplasia.

[8] Zhu J, Zheng Y, Liu Y, Chen M, Liu Y, Li J (2023). Association between HMGA1 and immunosuppression in hepatocellular carcinoma: a comprehensive bioinformatics analysis. Medicine (Baltimore).

[9] Xiao Y, Yu D (2021). Tumor microenvironment as a therapeutic target in cancer. Pharmacol Ther.

[10] Jiang Y, Zhang H, Wang J, Liu Y, Luo T, Hua H (2022). Targeting extracellular matrix stiffness and mechanotransducers to improve cancer therapy. J Hematol Oncol.

[11] Jhunjhunwala S, Hammer C, Delamarre L (2021). Antigen presentation in cancer: insights into tumour immunogenicity and immune evasion. Nat Rev Cancer.

[12] De Martino D, Bravo-Cordero JJ (2023). Collagens in cancer: structural regulators and guardians of cancer progression. Cancer Res.

[13] Liu XL, Ding J, Meng LH (2018). Oncogene-induced senescence: a double-edged sword in cancer. Acta Pharmacol Sin.

[14] D’Ambrosio M, Gil J (2023). Reshaping of the tumor microenvironment by cellular senescence: an opportunity for senotherapies. Dev Cell.

[15] Olan I, Ando-Kuri M, Parry AJ, Handa T, Schoenfelder S, Fraser P (2024). HMGA1 orchestrates chromatin compartmentalization and sequesters genes into 3D networks coordinating senescence heterogeneity. Nat Commun.

[16] Chen J, Ji K, Gu L, Fang Y, Pan M, Tian S (2022). HMGA1 promotes macrophage recruitment via activation of NF-κB-CCL2 signaling in hepatocellular carcinoma. J Immunol Res.

[17] Li X, Yao W, Yuan Y, Chen P, Li B, Li J (2017). Targeting of tumour-infiltrating macrophages via CCL2/CCR2 signalling as a therapeutic strategy against hepatocellular carcinoma. Gut.

[18] Eggert T, Wolter K, Ji J, Ma C, Yevsa T, Klotz S (2016). Distinct functions of senescence-associated immune responses in liver tumor surveillance and tumor progression. Cancer Cell.

[19] Hutter C, Zenklusen JC (2018). The cancer genome atlas: creating lasting value beyond its data. Cell.

[20] GTEx Consortium (2020). The GTEx Consortium atlas of genetic regulatory effects across human tissues. Science.

[21] Chandrashekar DS, Karthikeyan SK, Korla PK, Patel H, Shovon AR, Athar M (2022). UALCAN: an update to the integrated cancer data analysis platform. Neoplasia.

[22] Uhlen M, Zhang C, Lee S, Sjöstedt E, Fagerberg L, Bidkhori G (2017). A pathology atlas of the human cancer transcriptome. Science.

[23] Wei YG, Yang CK, Wei ZL, Liao XW, He YF, Zhou X (2022). High-mobility group AT-hook 1 served as a prognosis biomarker and associated with immune infiltrate in hepatocellular carcinoma. Int J Gen Med.

[24] Liu L, Zhang S, Hu L, Liu L, Guo W, Zhang J (2017). HMGA1 participates in MHCC97H cell proliferation and invasion through the ILK/Akt/GSK3β signaling pathway. Mol Med Rep.

[25] Kim KH, Roberts CW (2016). Targeting EZH2 in cancer. Nat Med.

[26] Cartwright T, Perkins ND, C LW (2016). NFKB1: a suppressor of inflammation, ageing and cancer. FEBS J.

[27] Rashid M, Rostami Zadeh L, Baradaran B, Molavi O, Ghesmati Z, Sabzichi M (2021). Up-down regulation of HIF-1α in cancer progression. Gene.

[28] Narita M (2007). Cellular senescence and chromatin organisation. Br J Cancer.

[29] Chen J, Li H, Huang Y, Tang Q (2024). The role of high mobility group proteins in cellular senescence mechanisms. Front Aging.

[30] De Martino M, Nicolau-Neto P, Ribeiro Pinto LF, Traverse-Glehen A, Bachy E, Gigantino V (2021). HMGA1 induces EZH2 overexpression in human B-cell lymphomas. Am J Cancer Res.

[31] De Martino M, Esposito F, Fusco A (2022). Critical role of the high mobility group A proteins in hematological malignancies. Hematol Oncol.

[32] Chang CJ, Hung MC (2012). The role of EZH2 in tumour progression. Br J Cancer.

[33] Concetti J, Wilson CL (2018). NFKB1 and cancer: friend or foe?. Cells.

[34] Cui X, Yun X, Sun M, Li R, Lyu X, Lao Y (2023). HMGCL-induced β-hydroxybutyrate production attenuates hepatocellular carcinoma via DPP4-mediated ferroptosis susceptibility. Hepatol Int.

[35] Seshacharyulu P, Halder S, Nimmakayala R, Rachagani S, Chaudhary S, Atri P (2022). Disruption of FDPS/Rac1 axis radiosensitizes pancreatic ductal adenocarcinoma by attenuating DNA damage response and immunosuppressive signalling. EBioMedicine.

[36] Yu DC, Liu J, Chen J, Shao JJ, Shen X, Xia HG (2014). GGPPS1 predicts the biological character of hepatocellular carcinoma in patients with cirrhosis. BMC Cancer.

[37] Grohmann M, Wiede F, Dodd GT, Gurzov EN, Ooi GJ, Butt T, et al. Obesity drives STAT-1-dependent NASH and STAT-3-dependent HCC. Cell 2018;175(5):1289-306.e20. doi: 10.1016/j.cell.2018.09.053. PMC6242467 30454647

[38] Tan S, Wang Z, Li N, Guo X, Zhang Y, Ma H (2023). Transcription factor Zhx2 is a checkpoint that programs macrophage polarization and antitumor response. Cell Death Differ.

[39] Dai DL, Xie C, Zhong LY, Liu SX, Zhang LL, Zhang H (2024). AXIN1 boosts antiviral response through IRF3 stabilization and induced phase separation. Signal Transduct Target Ther.

[40] Guerra B, Recio C, Aranda-Tavío H, Guerra-Rodríguez M, García-Castellano JM, Fernández-Pérez L (2021). The mevalonate pathway, a metabolic target in cancer therapy. Front Oncol.

[41] Kang H, Oh T, Bahk YY, Kim GH, Kan SY, Shin DH (2019). HSF1 regulates mevalonate and cholesterol biosynthesis pathways. Cancers (Basel).

[42] Chen Y, Lee D, Kwan KK, Wu M, Wang G, Zhang MS, et al. Mevalonate pathway promotes liver cancer by suppressing ferroptosis through CoQ10 production and selenocysteine-tRNA modification. J Hepatol. 2025. doi: 10.1016/j.jhep.2025.06.034. 40653112

[43] Wang K, Liu H, Hu Q, Wang L, Liu J, Zheng Z (2022). Epigenetic regulation of aging: implications for interventions of aging and diseases. Signal Transduct Target Ther.

[44] Zhang W, Hu D, Ji W, Yang L, Yang J, Yuan J (2014). Histone modifications contribute to cellular replicative and hydrogen peroxide-induced premature senescence in human embryonic lung fibroblasts. Free Radic Res.

[45] Sanders YY, Liu H, Zhang X, Hecker L, Bernard K, Desai L (2013). Histone modifications in senescence-associated resistance to apoptosis by oxidative stress. Redox Biol.

[46] Olan I, Narita M (2022). Senescence: an identity crisis originating from deep within the nucleus. Annu Rev Cell Dev Biol.

[47] Cheong JE, Sun L (2018). Targeting the IDO1/TDO2-KYN-AhR pathway for cancer immunotherapy - challenges and opportunities. Trends Pharmacol Sci.

[48] Löbrich M, Jeggo PA (2007). The impact of a negligent G2/M checkpoint on genomic instability and cancer induction. Nat Rev Cancer.

[49] Salminen A (2022). Role of indoleamine 2,3-dioxygenase 1 (IDO1) and kynurenine pathway in the regulation of the aging process. Ageing Res Rev.

[50] Kokubo K, Onodera A, Kiuchi M, Tsuji K, Hirahara K, Nakayama T (2022). Conventional and pathogenic Th2 cells in inflammation, tissue repair, and fibrosis. Front Immunol.

[51] Yang YM, Kim SY, Seki E (2019). Inflammation and liver cancer: molecular mechanisms and therapeutic targets. Semin Liver Dis.

[52] Gu FM, Li QL, Gao Q, Jiang JH, Zhu K, Huang XY (2011). IL-17 induces AKT-dependent IL-6/JAK2/STAT3 activation and tumor progression in hepatocellular carcinoma. Mol Cancer.

[53] Sun D, Li W, Ding D, Tan K, Ding W, Wang Z (2024). IL-17a promotes hepatocellular carcinoma by increasing FAP expression in hepatic stellate cells via activation of the STAT3 signaling pathway. Cell Death Discov.

[54] Taghizadeh Anvar M, Rashidan K, Arsam N, Rasouli-Saravani A, Yadegari H, Ahmadi A (2024). Th17 cell function in cancers: immunosuppressive agents or anti-tumor allies?. Cancer Cell Int.

