# Supplementary material for: Prognostic Significance of HMGA1 in Hepatocellular Carcinoma: Implications for Tumor Progression and Targeted Therapy
Source: Arch Iran Med. 2025 Oct 1;28(10):568–83. doi: 10.34172/aim.34456 (PMC12958422; doi:10.34172/aim.34456)
Supplement: Supplementary file 1 — contains Figures S1-S3. [file aim-28-568-s001.pdf]

Arch Iran Med. October 2025;28(10):568-S1

10.34172/aim.34456

http://journalaim.com

Supplementary file 1

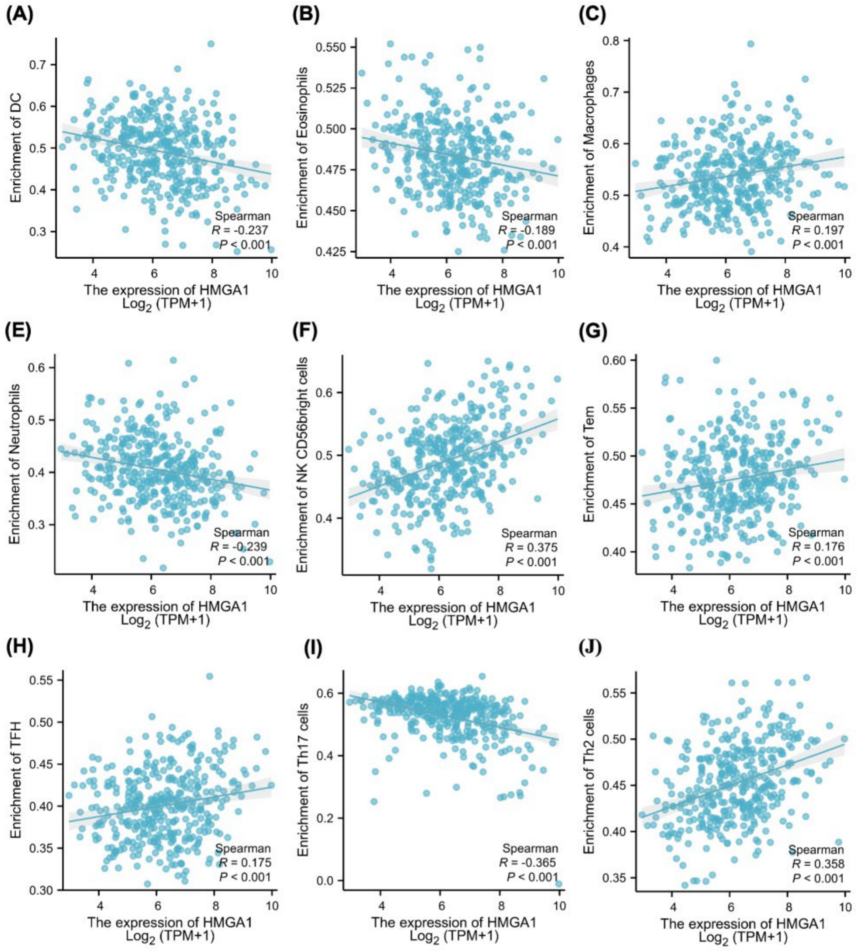

Figure S1. Scatter plots of the relationship between HMGA1 expression and immune cell infiltration scores, with statistically significant correlations ($p < 0.001$).

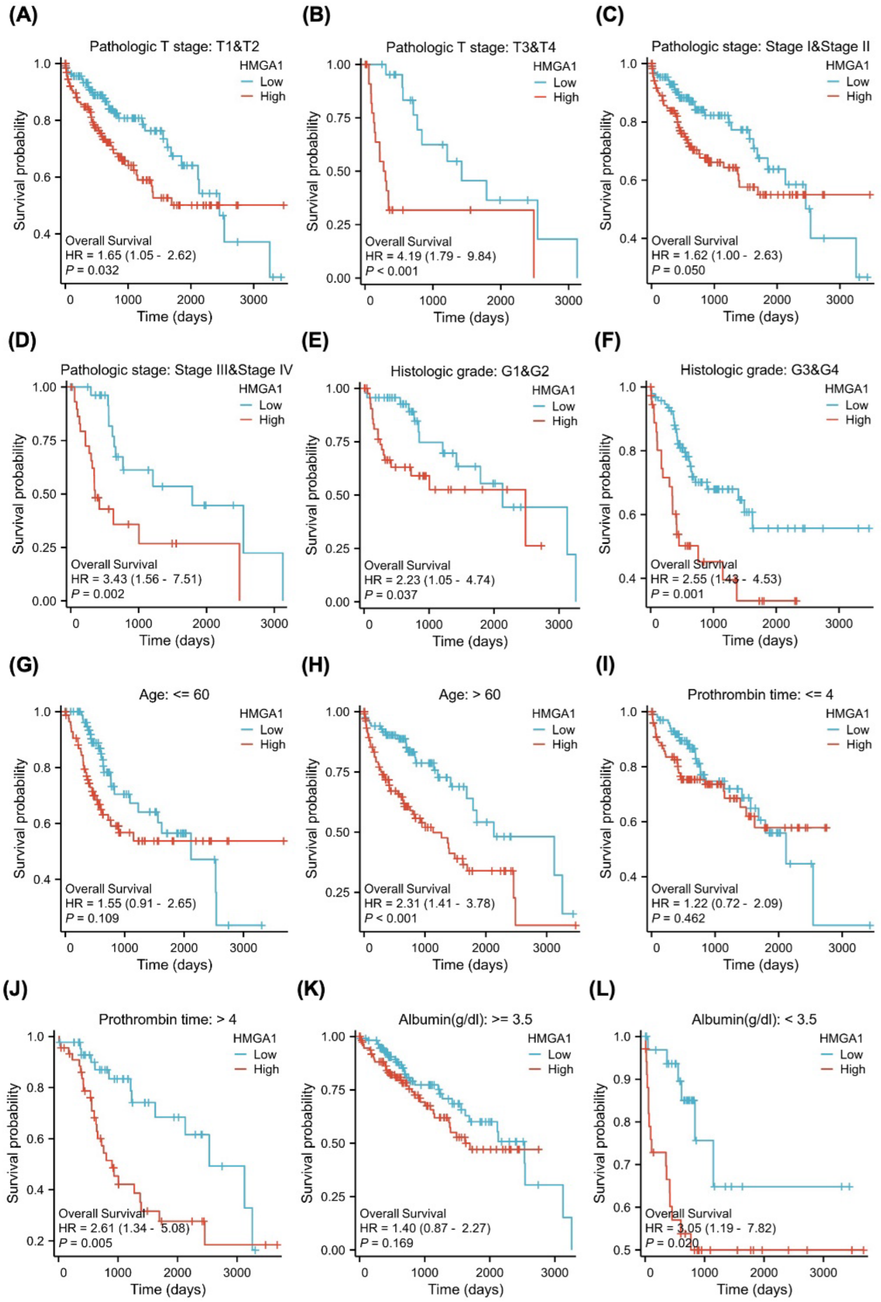

Figure S2. Results of survival analysis for different subgroups. Showing that higher HMGA1 expression is associated with poorer survival outcomes.

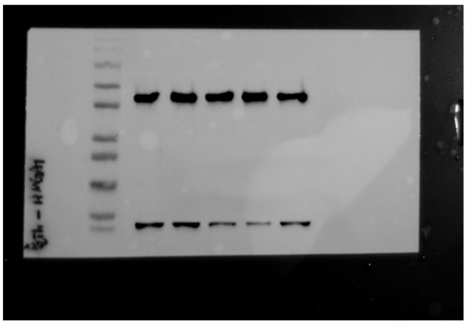

HepG2

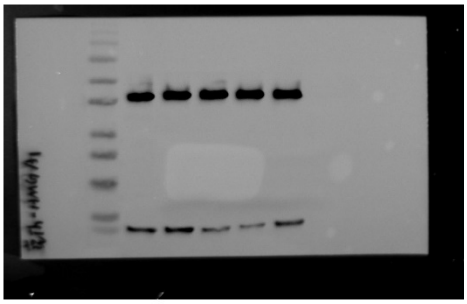

Huh-7

Figure S3. Original blots in Western blot analysis
